# Incorporating Systems Science Principles into the Development of Obesity Prevention Interventions: Principles, Benefits, and Challenges

**DOI:** 10.1007/s13679-015-0147-x

**Published:** 2015-04-09

**Authors:** Joel Gittelsohn, Yeeli Mui, Atif Adam, Sen Lin, Anna Kharmats, Takeru Igusa, Bruce Y. Lee

**Affiliations:** Global Obesity Prevention Center (GOPC), Johns Hopkins University, Room 2041A, 615 North Wolfe St, Baltimore, MD 21205-2179 USA

**Keywords:** Obesity, Prevention, Systems science models, Urban, African American, Principles, Benefits, Challenges

## Abstract

Systems modeling represents an innovative approach for addressing the obesity epidemic at the community level. We developed an agent-based model of the Baltimore City food environment that permits us to assess the relative impact of different programs and policies, alone and in combination, and potential unexpected consequences. Based on this experience, and a review of literature, we have identified a set of principles, potential benefits, and challenges. Some of the key principles include the importance of early and multilevel engagement with the community prior to initiating model development and continued engagement and testing with community stakeholders. Important benefits include improving community stakeholder understanding of the system, testing of interventions before implementation, and identification of unexpected consequences. Challenges in these models include deciding on the most important, yet parsimonious factors to consider, how to model food source and food selection behavior in a realistic yet transferable manner, and identifying the appropriate outcomes and limitations of the model.

## Introduction

Since the multifactorial causes and nature of obesity is well recognized, it is clear that no single community program or policy is likely to offer a comprehensive solution [1, 2]. Therefore, there is a need for multilevel, multicomponent (MLMC) interventions in the community to address obesity [3]. Such interventions, which work in many community settings simultaneously (e.g., food stores, schools, restaurants, worksites, etc.) have been shown to successfully prevent obesity [4–6]. However, MLMC interventions can be costly and challenging to design, implement, and evaluate. Communities are complex systems and interventions must plan for these complexities. Without adequate assurances, decision makers may be reluctant to try a MLMC intervention.

In many other fields, computational modeling has helped decision makers address a variety of different problems that sit in complex systems. For example, computational modeling is routinely used to help design, build, and evaluate transportation systems for cities and regions [7]. Many companies utilize simulation modeling to plan their manufacturing operations [8]. In the healthcare arena, simulation modeling has helped plan vaccine distribution and the prevention and control of infectious diseases in healthcare facilities [9–11].

What then is the potential of computational models in helping design, implement, and evaluate community-level obesity prevention and control measures? We review how computational models have been used for such interventions, use our ongoing obesity prevention trial to show the steps and key principles entailed in such applications of computational modeling, and summarize the benefits, challenges, and potential future directions of this work.

## Brief Review of Obesity-Related Modeling

Compared to the use of modelling in infectious disease research, computational modeling for community-level obesity control interventions is nascent. Therefore, many of the existing models are explanatory, attempting to elucidate the mechanisms behind obesity. Some have focused on mechanisms at the genetic and physiology scales as well. For example, a model developed by Butte et al. looks at the physiological processes and energy balance within individuals that produce obesity in individuals [12]. Other recent work has aimed to delineate individual or small group behavior that produces increased obesity risk [13]. At the opposite end of the spectrum, some policy models represent very broad effects at a national level. There have been few attempts to tie these different mechanistic scales into one cohesive model. The Foresight program of UK published an obesity system map, illustrating 108 variables related to obesity and their interconnected relationships [14]. This map has been further simplified by other researchers [15, 16]. While all of these models have been helpful in elucidating mechanisms, they have not been oriented to impact and evaluate community-level decision-making and operations.

One form of modeling, agent-based models (ABMs) could help develop solutions to the obesity epidemic [17•]. ABMs include computational “agents” that can represent a person or group that act autonomously and demonstrate adaptive behavior within a virtual representation of a location or region [18]. Each agent is assigned characteristics (e.g., age, gender) and a set of behaviors and decision-making rules for interacting with other agents and the simulated environment [19••]. In the obesity field, ABMs have been developed to evaluate the effects of social norms on diet and physical activity [20] as well as the relationships between income inequalities, segregation, and healthy eating [19••]. Computational modeling has also been used to assess the effects of economic change [21] and social networks [22] on the obesity epidemic.

## Developing and Utilizing a Computational Model for Community-Level Decision-Making: A Case Study in Baltimore City

To illustrate the steps, processes, and considerations involved in developing a computational model that can assist community-level decision-making, we will provide the example of the development and use of our Baltimore Low Income Food Environment (BLIFE) model, a geospatially specific agent-based model (ABM) representing low-income African American adolescents and their after school food environment in low-income neighborhoods in Baltimore City. Development of BLIFE occurred in Netlogo, a multiagent programmable modeling environment [23]. Using this model as an example, we will address the following questions:

1. What key principles underlie the development of multilevel simulation models to address the obesity epidemic?
2. What are the potential benefits of these models?
3. What are some of the most significant challenges that must be addressed when building systems science models for obesity prevention, and how might they be overcome?

## Key Principles

We have identified five principles for systems science modeling for community-based obesity prevention:

### Principle 1: Understand and Engage with the Community Being Modeled Prior to Developing the Model

To be relevant to decision makers for a community, it is important to have a deep understanding of and connection with the community. The groundwork for BLIFE was laid long before development of the ABM began. Members of the BLIFE development team have had over 12 years of experience in improving the Baltimore City food environment, through multiple intervention trials in corner stores [24–26], carryouts [27, 28], recreation centers [29], and churches [30, 31]. Additionally, the team has strong, longstanding connections with key stakeholders in Baltimore City, including the City Health Department, City Council, Department of Recreation and Parks, Food Policy Advisory Committee, and more.

### Principle 2: Integrate Model Development with Data Collection

BLIFE development was tied to extensive data collection activities. BLIFE includes a grid for mapping homes, schools, food sources (i.e., corner stores, carryouts, supermarkets), and recreation centers, covering approximately 15 % of the total area of Baltimore City in its current version. The model incorporates Maryland Food System Data from the Center for the Livable Future [32] and baseline data from the B’more Healthy Communities for Kids Program (BHCK). BHCK data were collected from 299 low-income African American children ages 10 to 14 and their caregivers living in food deserts [33••]. In addition to anthropometric measurements, the youth completed a questionnaire that assessed food-purchasing behavior, attitudes about healthy and unhealthy food, self-efficacy for making positive changes to their diet, and food-related psychosocial factors. They were asked how much money they typically spend when they buy food from carryouts and corner stores and how often they shopped in corner stores, carryouts, and fast-food restaurants, convenience stores, supermarkets, or at schools or other locations in the last 7 days.

### Principle 3: Represent All of the Processes That May Be Relevant to the Interventions Under Consideration, but No More

The principle of parsimony is important in modeling. The model should not include excessive detail that can cloud relevant interactions and relationships. However, the model should include every process that is relevant to the interventions being explored. The BLIFE model captures adolescent behaviors (food foraging and physical activity) on a daily basis. Agents in the model are characterized based on real data that inform their movement from school to small food store(s) to recreation centers to home. Decision-making of agents in the simulated food environment was based on data collected from 299 African American adolescents (10–14 years). Growth for each agent in the ABM is simulated over a 5-year period, and the weight trajectory of each agent is a perturbation of the CDC growth charts [33••, 34]. As the model is geospatially specific, food foraging and activity behaviors vary based on the agent and environment characteristics. At each time period, the agent computes the likely food source it accesses or the recreation center it could go to. Our studies have shown that afterschool consumption at small food stores is a major source of added calories and this has been incorporated in the model to make it more locally relevant. On the other hand, purchase and consumption of foods from fast-food restaurant chains is not common among our sample of children and is not represented in the model—invoking the principle of parsimony.

### Principle 4: Involve Stakeholders in Model Development to Give Them “Ownership” of the Model

We found that grounding the model in data from the specific communities involved helped convince decision makers that the model truly represents their own communities. Stakeholders are more likely to trust a model if they understand and feel ownership. This can be achieved by involving them along each step of the model development process. For BLIFE, we conducted multiple meetings with groups of policymakers and city agency representatives, where they were given the opportunity to review the model, suggest means of improving it and making it more relevant to their work.

### Principle 5: Make the Model Understandable and Accessible to Key Decision Makers

Stakeholders are less likely to trust a model that is a “black box” to them and that they cannot “test-drive” themselves. To help facilitate the accessibility of BLIFE, we developed a graphical user interface (GUI) for BLIFE. This GUI allows the user to modify characteristics of the simulated food environment that can influence dietary behaviors and obesity risk through the use of several “levers.” These were determined based on findings from the literature and from our extensive intervention work to improve the food environment in Baltimore.

The BLIFE model has generated considerable interest among local policymakers, city agencies, and other community institutions, who view it as a means of providing evidence in support of new and ongoing policy initiatives and testing what-if scenarios to examine the potential intended and unintended consequences of new programs. For example, the Family League of Baltimore, anticipating proposed closures of many of its summer meal program sites, found utility in the model to predict potential impact on household food security. Other city stakeholders have asked us to use the BLIFE model to simulate the effects of programs and policies with many diverse outcomes, including changes to food access, school achievement, and the financial viability of small food stores associated with new Farm Bill regulations.

## Benefits of Modeling for Communities

We have identified several benefits of ABM from the research and community perspectives:

### Benefit #1: Helps Community Stakeholders Understand How Multiple Variables, Factors, and Interventions Interact

Without the assistance of computational models, humans can struggle to consider more than a few factors and relationships at a time—and the potential consequences may be unclear. The BLIFE model, for example, weighs multiple factors when considering where a youth goes to purchase food, including proximity, type of food source, previous reported use of food sources, and relationship to school and home locations.

### Benefit #2: Test Potential Strategies Before Going to Scale, Saving Time and Money

Simulation modeling can test the potential impact of programs and policies in the “safety” of a virtual environment before they can be implemented, saving considerable time, effort, costs, and resources. Findings from simulation models can provide insight on the most promising approaches, when presented with a number of options. For example, the BLIFE model has been used to test the expected change in adolescent BMI percentile, over a 5-year period, as a result of (1) improving the availability of healthy food options in corner stores alone, (2) improving the availability of healthy food options in carryout restaurants alone, and (3) improving the availability of healthy food options in both corner stores and carryouts [35].

### Benefit #3: Demonstrates Potential Secondary and Tertiary Effects (and Even Unintended Consequences) of Intervention Strategies

A continuing challenge of working with MLMC interventions is that any action may have ripple effects throughout different parts of the system. Traditional methods that focus on only a component or portion of the system may miss these reverberations. From the BLIFE model work, we have come to realize that small stores can only access the foods that exist in their own food environment (what is offered by their wholesalers). Requiring these small stores to stock a broad range of healthy foods without working at higher levels of the food system to improve access could have the consequence of making these small businesses unworkable.

### Benefit #4: Can Guide/Prioritize Data Collection

Data collection is time consuming and expensive. It can be difficult to determine the impact of having certain types of data. Simulation modeling can help determine the value of having different types of data and can help prioritize selection of data sources. We have found it crucial to understand the food selection process of youth and how that might or might not be impacted by different intervention strategies (e.g., labeling, reducing price, increasing availability).

### Benefit #5: Facilitates Dialogue Among Stakeholders

A model can help communicate a person’s or group’s understanding of a system. In many ways, a model puts this understanding on the “table” for key stakeholders to consider, evaluate, critique, and discuss. It gives stakeholders something to react to and galvanizes discussion. For example, based on the request from a local city council member, the BLIFE model was used to simulate the potential impact of implementing a city tax credit for urban farmers on improving adolescent access to fresh produce and dietary behavior. The research team was invited to present findings from the simulation in support of the tax credit at a public hearing session held in October 2014.

## Challenges Encountered

In developing the BLIFE model, we have encountered multiple challenges.

### Challenge #1: Choosing What Factors and Relationships to Include or Exclude

A model is by definition a simplification and cannot possibly include every possible factor and relationship. A factor or relationship should be included if it may affect the ultimate evaluation or outcome of the intervention. In some cases, a factor or relationship is included if a key stakeholder would like to see it even though it has little impact on the results. As complete consensus might be harder to reach on some points, choosing what factors and relationships to not include requires judgment and experience. For example, the current version of the BLIFE model does not include social networks and peer relationships as a driving force in food selection. This is largely due to the complexity in collecting these data. We have used formative research as part of the process in determining key components to operationalize. Formative research not only helps in planning and development of intervention strategies and data collection instruments [36] but can also be used to identify the most salient and important factors for inclusion in simulation models. For example, our formative research indicated that corner stores were commonly used by children in low-income areas of Baltimore—which led us to carefully operationalize this component of the food system [33••]. On the other hand, carryouts are much less frequently used by youth and play a smaller role in their diet.

### Challenge #2: A Model Should Be Used for the Purpose That It Was Designed

Once a model is developed, there is the temptation to use it for every question or decision that arises. However, every model has its limitations, which should be clearly stated. The current iteration of the BLIFE model evaluates different obesity prevention programs and policies alone or in combination—in terms of its impact on childhood obesity, and cannot be used to assess economic effects.

### Challenge #3: How Best to Incorporate and Operationalize/Quantify Key Relationships/Feedback Loops?

A key feature of obesity epidemic is that there exist multilevel interactions among the different components. For instance, store owners adjust stock and price according to children’s purchase behavior and policy intervention, while children choose foods based on price, availability, and promotion. Store owners interact with wholesalers in a similar kind of supply-demand loop. These feedback loops can be modeled using behavioral algorithms to capture the interaction between agents in an ABM. The current BLIFE model does not contain feedback loops. This weakness exists due to several factors. Foremost is the enormous number of feedback loops involved in modeling food selection behavior over time. Each time a child visits a store and selects a kind of food, there will be feedback impacting the decision-making moving forward. Hence, the whole system will have a very large number of feedback loops for each combination of child, store, and food. Another challenge is that individual-level behavioral algorithms cannot capture all store-level changes. Selling of cold beverages strongly depends on daily weather, while many other foods do not. Fresh fruit stocking is highly seasonal. Customers of stores near schools will not visit during holidays, even if the prices are low. To model the feedback loops reasonably, surveys have to be conducted across multiple components of the model to effectively capture food stocking and purchases.

### Challenge #4: To What Degree Should Data on Current Practices (Behaviors) Be Incorporated into Modeling?

A key challenge is the degree to which actual behavioral data should be used to simulate decision-making. For example, for the BLIFE model, we used survey data on store selection and food purchasing by youth to create relative proportions of selection of different kinds of stores and foods. This makes the model very accurate (presumably) for predicting food-purchasing behavior in the Baltimore setting. On the other hand, there is a potential loss of transferability and generalizability from the model to other settings. Data on food store selection and purchasing are very specific to a particular setting and are related to the availability of those foods, their prices, amount of money youth in that setting have in hand, etc. It is important to come up with more general algorithms for modeling food choices (and other behaviors).

### Challenge #5 (Food Sources): How to Model Selection of Food Sources?

Using proximity as a sole measure of food store selection provides inconsistent findings when linked to dietary intake and obesity [37–48]. A more comprehensive understanding involves incorporating multiple personal, socio-environmental, and behavioral factors [49, 50]. The dynamics of these influences are very difficult to understand through statistical models alone, hence, the utility of ABMs. In silico models developed by Auchincloss et al. showed how residential income segregation at the community level can impact dietary behavior by disaggregating the distribution of grocery stores [19••]. The model highlighted that spatial segregation among high-income and low-income areas can exacerbate residential availability of healthy foods in those areas. Social influences on eating behaviors have been explored in ABM by Zhang et al. [51]. For the BLIFE model, the agents take advantage of the extensive data collected previously to capture food source selection in Baltimore City based on food-purchasing behavior, neighborhood availability, attitudes about healthy and unhealthy food, etc. (detailed earlier).

### Challenge #6: What Are the Factors That Influence Food Choice Decisions of Youth and Other Key Actors Within a Selected Food Source?

A range of factors influences adolescent food choice within a store. Food choices can be influenced by promotional signage, displays, and taste-tests in stores [33••]. Structural changes in stores (i.e., refrigeration units) and storeowner trainings can encourage consumer purchasing of healthier food options [52]. Formative research from the BHCK obesity-intervention trial further revealed that taste, preference, and familiarity with a given food were important factors in decision-making [33••]. For example, some found that diet beverages were not as satisfying or palatable as regular sugar-sweetened beverages. Another factor is price sensitivity of adolescents, especially in urban low-income neighborhoods. Reduction in pricing has been shown to influence food choice as well [28]. Finally, while identifying the most important drivers is challenging, system dynamics modeling and social network analyses can be helpful to tease apart the influence of parental and household factors (i.e., income, education) and to better understand the underlying mechanisms of food choice. For the BLIFE model, we developed an algorithm to model food choice based on reported frequency of consumption of foods from different categories, classified as healthy and unhealthy options. For example, our data indicated that children were three times more likely to select a sugar-sweetened beverage than a healthier alternative like water.

### Challenge #7: How Should Physical Activity Be Incorporated into Obesity Prevention Models?

Changes in BMI are reflective of the energy imbalances between energy intake/storage and mobilization (energy expenditure, growth) [53]. The factors that influence energy expenditure and energy intake have varying degree of influence and complexity [54, 55]. A model developed by Butte et al. has been widely used in systems modeling literature to predict changes in energy intake and physical activity associated with weight gain in children and adolescents [12]. A modified version of the model has been used in the BLIFE model to understand energy imbalance in adolescents taking into account walking and recreational center activities. The next iteration of the BLIFE model will look to incorporate more types of physical activities to represent varying activity intensity levels (light, moderate, vigorous) [56].

### Challenge #8: How to Best Incorporate Potential Intervention Levers?

The usefulness of ABMs is directly related to how readily they can be employed to simulate the impact of different intervention strategies. For example, a lever which allows the user to change availability of healthy foods in small food stores would permit the testing of this strategy. Many such levers could be conceptualized, such as pricing, promotional materials, training, etc. However, the addition of more levers means more increased complexity and challenges for how to weigh each of these additional variables. Should the impact of lowered prices for healthier foods be weighted as more influential than materials (like shelf labels) promoting the health benefits of these foods? For the BLIFE model, we developed an array of over 30 different levers, with 3–6 levers assigned to each component of the food environment (corner stores, carryouts, etc.).

### Challenge #9: Need for Integration of Social Networks into Complex Systems Models?

Previous, obesity-related ABMs lacked an empirically based social network structure. The agents often moved randomly in simulated environments or develop a network randomly in their environment with other agents based on a limited set of requirements [57•, 58]. Similarly, studies of social networks and their relationship to obesity have not rigorously investigated the interactions between the environments in which children live, study, and play and how these environments shape social networks and are in turn shaped by them [22, 59, 60]. Instead, researchers have focused on defending whether the environment or social networks are more important in explaining the “spread” and clustering of obesity over time and controlled for the other variables. This work is complicated by a lack of method operationalization and standardization [61]. These measures are only weakly associated with obesity prevalence and do not actually reflect how individuals interact within their local food environments [39, 62]. The BLIFE model adds to other recent advances in the social environments and obesity literature [63, 64] by using data from real children, precisely identifying where children are obtaining food, how often, and with whom. This specificity will help us investigate the interaction between food environments and social networks as they relate to clustering of obesity in social networks and neighborhoods.

### Challenge #10: What Are the Most Appropriate Outcomes to Track?

Development of any systems science model should be driven by a specific inquiry—what is the purpose of developing the model and what question is one seeking to answer with the model? For the BLIFE model, the outcome of interest was change in youth BMI percentile and obesity risk. However, there is significant value added when stakeholders and policymakers can utilize the evidence from systems science models to inform their own programs and policies. Therefore, it is important to consider other outcomes that may be of interest to a range of users. We have been asked to expand the BLIFE model to be used as a tool to examine potential change in student attendance/student achievement, energy intake/energy expenditure, and food security and hunger.

## Conclusions

Simulation modeling has tremendous potential to transform the design, planning, implementation, and evaluation of community-level obesity control. The use of modeling in this realm is still relatively nascent as much of the existing obesity-related models have been focused on particular scales and purposes. We have outlined several guiding principles for developing community-level models for obesity control and highlighted the benefits and challenges involved in the process. As the use of simulation modeling to guide such interventions grows, stakeholders may become more accepting of this method. Greater experience will undoubtedly lead to modification of these principles, benefits, and challenges over time.

Additional research is needed to address each of the challenges listed above. It would help to develop a “shared language” among diverse stakeholders around obesity to encourage consensus building over time. Nevertheless, the benefit of being able to test potential interventions virtually before implementation to assess cost-effectiveness and potential impact is enormously valuable.

Importantly, it should be emphasized that computational models, such as the BLIFE agent-based model, should not replace human decision-making or other types of studies. Models are intended to facilitate human decision-making and complement other studies, but not replace them.
